# Aggressive Central Nervous System Relapse after Autologous Stem Cell Transplant in Multiple Myeloma: Case Reports and Literature Review

**DOI:** 10.1155/2020/8563098

**Published:** 2020-01-03

**Authors:** Rui Bergantim, Juliana Bastos, Maria José Soares, Bruno Carvalho, Pedro Soares, Cristina Marques, Jennifer Costa, José Eduardo Guimarães, Fernanda Trigo

**Affiliations:** ^1^Hematology Department, Centro Hospitalar São João, Porto, Portugal; ^2^i3S—Instituto de Investigação e Inovação em Saúde, Universidade do Porto, Porto, Portugal; ^3^Cancer Drug Resistance Group, IPATIMUP—Institute of Molecular Pathology and Immunology of the University of Porto, Porto, Portugal; ^4^Hematology & Oncology Unit, Department of Medicine, Faculty of Medicine of University of Porto, Porto, Portugal; ^5^Cytogenetics Laboratory, Hematology Department, Centro Hospitalar São João, Porto, Portugal; ^6^Neurosurgery Department, Centro Hospitalar São João, Porto, Portugal; ^7^Radiotherapy Department, Centro Hospitalar São João, Porto, Portugal; ^8^Flow Cytometry Laboratory, Clinical Pathology Department, Centro Hospitalar São João, Porto, Portugal; ^9^Pathologic Anatomy Department, Centro Hospitalar São João, Porto, Portugal

## Abstract

Extramedullary disease is an aggressive presentation at diagnosis and relapse for multiple myeloma (MM) patients. Central nervous system (CNS) is a very rare manifestation of the extramedullary disease, accounting for less than 1% of MM on diagnosis and relapse. Neurological symptoms are unspecific and usually attributed to other causes. We present two patients with CNS-MM at relapse after autologous stem cell transplant highlighting the importance of clinical suspicion and interdisciplinarity at diagnostic workup as well as the need for intensive therapeutic options on such rare and aggressive cases. The presence of neurological abnormalities in anamnesis and physical examination on a patient with MM should always prompt to suspect of a CNS involvement, and active investigation must be undertaken. MRI is the standard radiological method to detect CNS-MM, with histopathological corroboration by stereotactic biopsy and CSF evaluation alongside. Treatment of CNS-MM should include two essential approaches—be able to cross the BBB and treat the systemic disease. There is no standard therapy for this extramedullary relapse, and a tailored and multiple therapy should be promptly started—intrathecal therapy, radiotherapy, and systemic therapy, including an immunomodulator.

## 1. Introduction

Despite novel agents and increased better outcomes over the last years, multiple myeloma (MM) is still an incurable disease marked by a relapse-remission pattern [1]. Mainly characterized as a medullary monoclonal proliferation, some cases present as extramedullary disease. At diagnosis, the extramedullary disease is found in 6 to 8% of patients [2, 3], while another 10 to 30% may develop extramedullary lesions later in their disease course [4, 5]. Central nervous system (CNS) is a very rare manifestation of the extramedullary disease, accounting for less than 1% of MM on diagnosis and relapse [6, 7]. CNS involvement is defined by the presence of monoclonal malignant plasma cells in the cerebrospinal fluid or by magnetic resonance imaging revealing the bone-derived contiguous intraparenchymal mass, isolated intraparenchymal tumors, and leptomeningeal involvement [7–9]. Neurological symptoms are unspecific and usually attributed to other causes [8]. CNS-MM confers a dismal prognosis, with median survival of less than six months, being a final event in the majority of these patients [9, 10]. Approach and therapeutic options are not established for these patients, and many data rely on case reports and small series [9, 11, 12]. We present two patients with CNS-MM at relapse after autologous stem cell transplant, highlighting the importance of clinical suspicion and interdisciplinarity at diagnostic workup as well as the need for intensive therapeutic options on such rare and aggressive cases.

## 2. Case 1

A 49-year-old man with no relevant past clinical history was diagnosed with MM IgG/lambda, Durie–Salmon stage IIIA, and International Staging System (ISS) stage III in August 2011. He presented with anemia and extensive lytic bone lesions. His bone marrow assessment showed 33% of abnormal plasma cells, CD38+ve, CD19−ve, CD56+ve, CD45−ve, and lambda+ve and no alterations on fluorescence in situ hybridization (FISH) of selected plasma cells. He was treated with bortezomib and dexamethasone (VD) for four cycles, achieving a very good partial response (VGPR), followed by stem cell mobilization with an intermediate dose of cyclophosphamide (4 g/m^2^) collecting enough cells for two grafts, and he underwent his first autologous stem cell transplant (ASCT), after high-dose melphalan (200 mg/m^2^) in January 2012. He maintained a VGPR at day 100 assessment.

In August 2014, he presented with severe back pain leading to a progression evaluation. He had new lytic bone lesions on the skull and lumbar vertebrae, hypercalcemia, anemia, and acute renal failure. A new bone marrow assessment was performed with 40% of abnormal plasma cells and no FISH abnormalities identified. He was treated with bortezomib, thalidomide, and dexamethasone (VTD) for four cycles reaching only a partial response (PR), followed by high-dose melphalan and was submitted to a second ASCT in January 2015. At day 100, he had a VGPR and was kept on observation.

**Figure 1:**
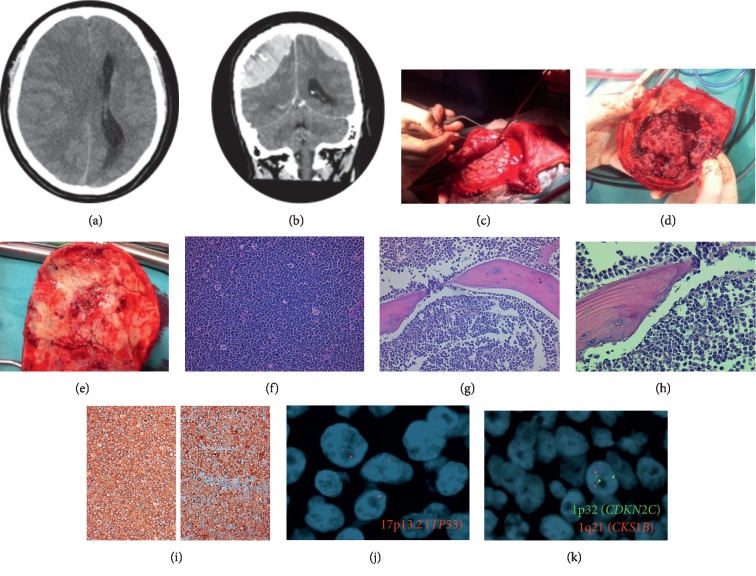
(a, b) Two massive extra-axial tissue lesions (right: 3.5 cm in the coronal plane; left: 1.4 cm in the coronal plane), spontaneously hyperdense and with strong contrast enhancement; posterior extension of the lesion with dura infiltration; diffuse involvement calvaria; (c–e) surgical removal of the frontoparietal bone flap with adherent soft tissue lesion and dura mater; (f–i) plasma cells with high mitotic activity and apoptotic bodies. (HE, 200x); invasion of the bone. (HE, 400x); (j, k) 1p32/1q21 and 17p13.2 (TP53) copy number changes evaluated by FISH: (j) (17p13.2, red), (k) (1p32, green and 1q21, red), (j) del17p13.2 (30%), and (k) 1q21 gain (72%).

Nine months after the second ASCT, he was admitted to the emergency room in a comatose status (Glasgow Coma Scale 9). His blood workup was normal without anemia, hypercalcemia, or acute renal failure. Brain computed tomography showed two large extra-axial lesions (a right one with 3.5 cm in the coronal plane and a left one with 1.4 cm in the coronal plane), spontaneously hyperdense and with strong contrast enhancement, a posterior extension of the lesion with dura mater infiltration, and diffuse involvement of the calvaria. Due to rapid neurological deterioration, the patient was operated by neurosurgery with resection of the right frontoparietal lesion with invaded dura and bone flap. The anatomopathological exam showed an extensive collection of plasma cells with high mitotic activity, multiple apoptotic bodies, and extension to the adjacent bone. FISH analysis of the excised cerebral mass showed del17p13.2 (72%) and del1q21 (30%). His bone marrow had no plasma cells, no FISH abnormalities, and no serum or urine monoclonal component, besides the serum immunofixation Ig/lambda and abnormal free light chain ratio (Figure 1). After recovery from surgery, he started DPACE (dexamethasone, 40 mg/day; cisplatin, 10 mg/m^2^/day; doxorubicin, 10 mg/m^2^/day; cyclophosphamide, 400 mg/m^2^/day; and etoposide, 40 mg/m^2^/day from days 1 to 4) completing 2 cycles, simultaneous cranial radiotherapy (RT) 40 Gy (2.5 Gy per day for 16 sessions and a photon energy of 6 Mv), and started lenalidomide plus dexamethasone until progression. He completed 14 cycles. Although he had never achieved a complete remission, there was full recovery of functional and cognitive status with a performance status ECOG of zero by the time he was on the second cycle of lenalidomide. He progressed in May 2017 with a new lumbar extramedullary disease and was put on daratumumab, bortezomib, and dexamethasone (DVD) and simultaneous lumbar RT (3 Gy per day for ten sessions and photon energy 6 Mv). He completed 10 cycles of DVD, progressing again with extensive bone disease and rapid deterioration of his performance and was referred to palliative care.

## 3. Case 2

A 66-year-old man with no relevant past clinical history was diagnosed with MM IgA/lambda, Durie–Salmon stage IIIA, and ISS stage III in December 2014. He presented with anemia, hypercalcemia, and extensive lytic bone lesions on skull, thoracic, and lumbar vertebrae and long bones. His bone marrow showed 40% of plasma cells, CD38+ve, CD138+ve, CD19−ve, CD56−ve, and CD45+ve, with del13q (87%). He was treated with bortezomib, thalidomide, and dexamethasone for four cycles, achieving a complete remission (CR). He was proposed for ASCT with stem cell collection after steady mobilization with G-CSF and underwent high-dose melphalan (200 mg/m^2^) followed by ASCT in May 2015. On day 100 evaluation, he remained in CR.

**Figure 2:**
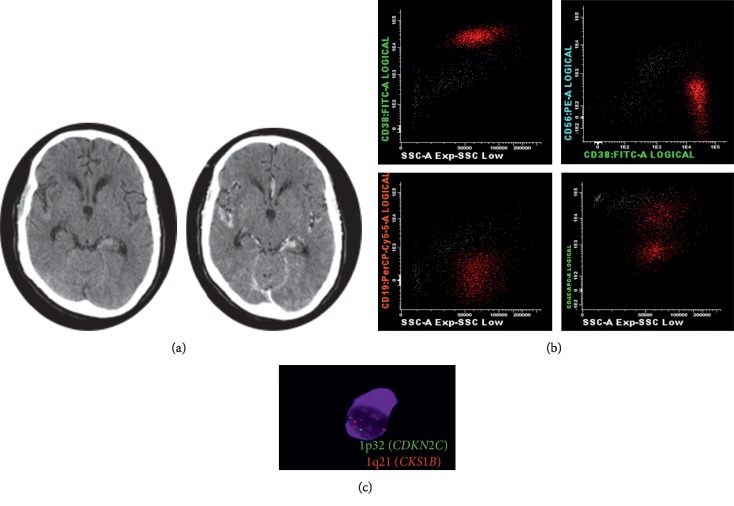
(a) Spontaneous hypodense areas with intense cortical contrast enhancement in bilateral insulae, anterotemporal predominance of the left grooves, engorgement of the left choroid plexus, right cerebellar tent, the right side of the prepontine cistern and pituitary gland, supratentorial ventricular dilatation with transependymal edema, focal area of lower density in the body of sphenoid, and extensive leptomeningeal infiltration. (b) 35 cells (87% neoplastic plasma cells). (c) 1p32/1q21 copy number changes evaluated by FISH: 1p32, green; 1q21, red and 1q21 gain (100%).

In November 2015, he went to the daycare hospital with refractory nausea, holocranial headaches, confusion, diplopia, and loss of muscle strength in the upper right limb with three days duration. His blood workup was normal without anemia, hypercalcemia, or acute renal failure. A cerebral scan was performed showing spontaneous hypodense areas with intense cortical contrast enhancement in the bilateral insula, anterior temporal predominance of the left sulci, engorgement of the left choroid plexus, right cerebellar tentorium, right prepontine cistern and pituitary gland, supratentorial ventricular dilatation with transependymal edema, extensive leptomeningeal infiltration, and focal low density on the sphenoid body. A lumbar puncture was performed with the cerebrospinal fluid (CSF) analysis showing 39 cells, of which 88.7% were monoclonal plasma cells CD38+ve, CD19−ve, CD56−ve, and CD45-het, with lambda restriction and all with del1q21 on FISH. His bone marrow had no plasma cells, no FISH abnormalities, and no serum or urine monoclonal component, besides the reappearance of serum immunofixation IgA/lambda and abnormal free light chain ratio (Figure 2). He received intrathecal therapy with methotrexate, cytarabine, and dexamethasone until plasma cell clearance that was achieved after 10 IT administrations followed by neuroaxial RT 16.5 Gy (1.5 Gy per day for 11 sessions with photon energy 6 MvM; this was suspended due to severe pancytopenia). Poor performance status at that point did not allow him to start systemic cytotoxic therapy, and he was treated with lenalidomide plus dexamethasone until progression. He completed ten cycles, with biochemical and imagiological criteria of CR and improved performance status, recovering his autonomy in most of all daily tasks. He progressed on September 2016 with new lumbar and cutaneous extramedullary disease and started daratumumab and dexamethasone, without response at the second cycle and deterioration of performance status being referred to palliative care.

## 4. Discussion

Both cases represented great challenges in clinical practice. No guidelines and recommendations are evident in how to approach or treat CNS-MM. In the past years, many reports suggest that extramedullary disease, including CNS-MM, increased with the use of novel agents and autologous stem cell transplant [4, 13]. So far, no plausible or physiopathological explanation supports this inference. Novel agents and ASCT are nowadays standard in clinical practice and better outcomes achieved with first-line therapies with longer disease-free survival and overall survival probably allow for appearance of new patterns of relapse and clinical manifestations [10, 14].

Clinical manifestations are usually unspecific and heterogeneous, like headaches and cognitive dysfunction, resembling other common neurological diseases or chemotherapy-related side effects making the differential diagnosis hard in clinical practice [7, 8]. The presence of neurological abnormalities in anamnesis or physical examination on a patient with MM should always prompt to suspect CNS involvement, and active investigation must be undertaken.

Contrast-enhanced MRI is the standard radiological method to detect CNS-MM, but sensitivity is lower for hematological malignancies (20–37%) than for solid tumors (85%) [15]. Moreover, patterns are diverse and difficult to interpret with most series accounting 10% of false negatives [6, 15–17]. Consequently, it is recommended to perform histopathological corroboration and CSF evaluation alongside, whenever possible. Stereotactic biopsy is not always possible due to location of infiltration, patient limitations, or the urgency of treatment initiation. Nonetheless, it should always be considered. The isolated presence of MM cells is not enough to assume CNS involvement, as in other hematologic diseases [16]. However, when it happens in a patient with MM that confers a high suspicion of effective CNS-MM. CSF protein is often increased while glucose is low on CNS-MM [7]. The recent use of abnormal free light chain detection on CSF is an attractive complementary diagnostic tool as it appears to be more reliable than cytology and could be used in patients with other equivocal test results and allowing therapy monitoring additionally [18–20]. The opposite is also possible, and patients have been described with intraparenchymal and dura lesions but with no plasma cells on CSF [17, 21]. Our two patients illustrate these disparities of findings. One had a massive involvement of CSF with a meningeal enhancement on MRI, but with no intracerebral mass to biopsy, while the other had an evident intraparenchymal infiltration with extension to dura mater and adjacent bone, evident on radiological studies, which was surgically approached. On clinical practice, all data available should be integrated to assure a reliable and safe diagnosis of CNS-MM.

There is no independent prognostic factor for the development of MM-CNS. Some reports show that the high-risk patients share some classic features related to adverse prognosis such as lambda and IgD subtype, high-burden disease, stage III by Salmon–Durie, high lactate dehydrogenase (LDH), and high beta2-microglobulin [7, 14, 22–24]. Extramedullary disease, rather than CNS, is often associated with the development of CNS-MM. Plasma cell leukemia (PCL) is a rare extramedullary MM characterized by >20% or 2000/mL monoclonal plasma cells in peripheral blood. Some series assume this as a significant factor for CNS involvement [7, 9]. More recently, the use of circulating plasma cells on peripheral blood also showed a higher burden disease and risk for adverse relapse [25]. Unfavorable chromosomal abnormalities are related to various extramedullary sites, indicating they may drive different clonal architecture between the bone marrow and extramedullary disease, mainly a possible role of p53 mutations/del17p13 [16, 26–28]. CD56 is an adhesion molecule involved in plasma cell migration from the bone marrow, and its downregulation could promote MM cells to escape their natural environment and establish a plasma cell metastasis at distance [16]. Chang et al. reported that the expression of CD56 was detected in 80% of patients with non-CNS-MM (*n*=84) and was absent in 62.5% of the bone marrow and 88% of CSF with CNS-MM (*n*=8) [29]. Nonetheless, other reports failed to demonstrate this [6]. Despite these risk factors, many cases of CNS-MM are characterized by a low degree of bone marrow infiltration [16], as in our 2 cases. The role of a CNS assessment in high-risk patients and concurrently prophylactic intrathecal chemotherapy is debatable, but it should be considered individually in the future patients.

There are some hypotheses that emerge as an explanation of CNS involvement by MM. The first hypothesis relates to the hematogenous spread in which plasma cells and earlier cells with a specific phenotype can induce metastasis to distant places as extramedullary disease including the CNS [14, 16]. In some series, the negativity of CD56 is appointed as a hallmark to CNS-MM, as its function is essential on the cell-cell adhesion as exposed previously [29, 30]. Bladé and colleagues in a report demonstrated that decreased expression of adhesion molecules on myelomatous cells over time would promote egress from the BM environment [4]. The presence of circulating plasma cells, and in an extreme phase as plasma cell leukemia, is also appointed to support this hypothesis. A report showed that 40% of PCL patients had some CNS involvement [9]. None of our patients had negativity to CD56 or circulating plasma cells. Autopsy studies in MM patients revealed that circulating plasma cells can infiltrate the arachnoid veins diffusely leading to the destruction of the arachnoid trabeculae and continuously spread to CSF [8, 16]. A second hypothesis, mainly in patients with parenchymal infiltration, is related to the extension of tissue infiltration of adjacent skull lytic lesions or plasmacytomas, varying from 39% to 65% in some cohorts [9, 14]. A third hypothesis relies on the continuous growth of plasma cell nesting in CNS, as the sanctuary site compared to lymphoblastic leukemia, during treatment, as most of the MM drugs used do not cross the brain-blood barrier (BBB) [7, 10]. Finally, the clonal heterogeneity of MM could play a role on this rare relapse, in which there is emergence of a clone population resistant to the treatment used on previous lines [14, 16, 31, 32]. Our patients showed high-risk features of the parenchymal tumor and CSF that were not present at diagnosis, hypothesizing that probably the relapse arose from a different clone rather than the one present at diagnosis.

Regarding the treatment of CNS-MM, significant data are sparse, and there is no standard of care advised for these cases [6, 9, 18]. Treatment of CNS-MM should include two essential approaches—be able to cross the BBB and treat the systemic disease.

Immunomodulators can cross the BBB. Thalidomide can be detected on CSF after an oral administration of 100 mg/day, peaking a concentration of 30% to 60% as compared to the plasma [33–35]. However, its neurological side effects, low efficacy on del17p patients, and the long time to an actual drug effect make its use less appealing. Lenalidomide and pomalidomide also showed to penetrate the BBB in animal studies [36–38]. Recent data showed that lenalidomide and pomalidomide have a moderate effect displaying a 30 to 40% penetration but enough to be effective in clearance of neoplastic cells from CSF in both lymphomas and myeloma [39–41]. This effect can also be seen with other disease markers and patients with POEMS exhibited a reduction of CSF VEGF when treated with lenalidomide as well as patients with astrocytoma, endorsing the use of IMIDs as antiangiogenic agents with the ability to cross the BBB [42].

Bortezomib has a limited penetration through the BBB in animal models, with few reports showing its inefficacy in CNS-MM patients [43], and there are no data on ixazomib. Marizomib is the only proteasome inhibitor known to be able to cross the BBB, even though it has so far not proved to be of use on MM. Radiolabeled marizomib showed 30% CNS biodistribution compared to blood levels on animal models. In a case report, two patients were treated with marizomib with reduction of CSF plasmacytosis as well as symptoms and had radiological improvement. Nonetheless, its activity in systemic myeloma is yet to be determined [44]. No precise data are supporting the use of daratumumab on CNS-MM, and the case report on marizomib also showed that the clearance of CD38 cells from CSF suggested that daratumumab may also cross the BBB and recently a case report showed a successful outcome with the monoclonal antibody [44, 45].

Intrathecal chemotherapy (ITC) is mostly used in several hematological malignancies with CNS involvement with diverging results [9]. The intrathecal agents are normally used in combination with systemic therapy that theoretically penetrates the BBB, and its use in monotherapy may be ineffective. Several reports show that patients that received ITC have a longer survival of 7.2 to 20 months versus 1.4 to 5 months for the patients who did not receive ITC [18, 19]. In Case 2, with myelomatous meningitis, no systemic therapy was administrated due to the patient's bad performance status. As a bridge to start chemotherapy, intrathecal administration of recognized agents is quickly done and usually fast on malignant cells clearance. Some series showed that ITC and IMIDs containing regimens had higher median survival comparing to IMID's monotherapy or other drugs (17.1 months versus 4.6 months) [9].

Brain and neuraxial radiation, as there is a well-known pattern of plasma cell radiosensitivity, seems an essential coadjuvant therapy in CNS-MM [9]. Nonetheless, it carries significant toxicity and its application may be limited to older patients and with a bad performance status. In the literature review, radiation was the only treatment option which showed to be associated with prolonged survival, presenting a median survival of 3 months versus 0.81 months (without radiation) [18]. Nonetheless, in other reports, RT improved survival was evident only when combined with new agents as a coadjuvant treatment [24].

Standard chemotherapy used on MM as alkylating agents penetrates the BBB very poorly, with only 10% of melphalan levels found at LCR of treated patients [7]. The intensive chemotherapy combinations such as PACE are useful as salvage regimens in refractory/relapsed MM and PCL and appear to be a robust approach to systemic treatment to prevent further hematogenous spread to CNS and elimination of residual clones [46]. As a sole treatment for CNS disease, it is insufficient. The combination with immunomodulators and proteasome inhibitors such as VTD (bortezomib, thalidomide, and dexamethasone)-PACE and VRD (bortezomib, lenalidomide, and dexamethasone)-PACE are attractive choices for this aggressive manifestation of MM [26, 35].

Our patients were treated according to presentation and performance status. In Case 1, the exuberance of presentation demanded a life-saving approach with the surgical approach, systemic therapy, radiotherapy, and immunomodulators. In Case 2, due to the absence of intraparenchymal tumor, the patient was treated with intrathecal chemotherapy, radiotherapy, and immunomodulators. We believe that the aggressive management of these patients followed by a continuous therapy with an agent that cross the BBB improved their survival comparing to published data.

Prognosis of CNS-MM is dismal across all series and case reports, with a post-CNS-MM survival of 3 to 6 months and does not appear to be different in patients treated with novel agents compared to those treated with conventional drugs [6, 7, 14, 18, 24]. In our cases, both patients had a surprising progression-free survival (PFS) of 14 and ten months, respectively, on Case 1 and 2. As prognostic factors are at relapse to post-CNS-MM survival, extramedullary disease and LDH continue to be strong predictors showing a tendency for more aggressive disease, demanding a closer follow-up [14]. The normal LDH and absence of other disease manifestations, including bone marrow infiltration, may influence the long PFS in our two patients.

These cases illustrate unusual patterns of CNS-MM involvement that could envisage two different hypotheses for CNS relapses such as the direct contiguous spread from the eroded lytic lesions of the skull (more probable in Case 1) and hematogenous spread of plasma cells (more probable in Case 2). Although rare, CNS involvement by MM should be considered in MM patients with neurological deficits/symptoms. There is no standard therapy for this extramedullary relapse, and a tailored aggressive therapy should be promptly started—ICT, RT, and systemic therapy, including an IMID. Even in the era of novel therapies, the prognosis of CNS involvement remains dismal, highlighting the need for adequate CNS penetration in MM novel drug development to improve these patients' outcomes.
